# Civic universities and bottom-up approaches to boost local development of rural areas: the case of the University of Macerata

**DOI:** 10.1186/s40100-021-00185-5

**Published:** 2021-08-03

**Authors:** Tomasi Sabrina, Cavicchi Alessio, Aleffi Chiara, Paviotti Gigliola, Ferrara Concetta, Baldoni Federica, Passarini Paolo

**Affiliations:** Department of Education, Cultural Heritage and Tourism, University of Macerata, P.le Bertelli, 1, 62100 Macerata, Italy

**Keywords:** Civic university, Rural areas, Quadruple Helix, Co-creation, Sustainable development

## Abstract

The aim of this paper is to provide a review of the main roles HEIs can play in rural areas. A longitudinal case study about the civic engagement of the University of Macerata - UNIMC (Italy) is presented, by assessing its attempt to fulfil its third and fourth mission through the application of the Quadruple Helix and 3 Model and by implementing the Civic University’s dimensions. Furthermore, these aspects have been investigated through the university-business collaboration and the community-academic-collaboration frameworks. More specifically, the paper focussed on UNIMC’s commitment at a local level analysing its involvement in local and international projects for place and agri-food product marketing, place branding and rural development, promoted by a research team within the Department of Education, Cultural Heritage and Tourism.

## Introduction

In the last decades, universities have become increasingly more engaged in the civic society, mainly in the rural areas where Higher Education Institutions (HEIs) have built relationships with local stakeholders and worked on co-creation activities to achieve sustainable development objectives (Trencher et al. 2013; Cavicchi et al. 2013). Many HEIs are directly involved and investigate the specific needs of local areas through place-based projects involving several actors in order to propose innovative solutions to real problems (Atterton and Thompson 2010; Ward et al. 2005; Trencher et al. 2014).

According to the Smart Specialisation Strategy (S3) promoted by the European Commission (Rinaldi et al., 2018), university plays a pivotal role in innovating the society as it becomes a means of cross-fertilisation and co-creation in different thematic areas and for different actors. It can support the achievement of sustainable development in the knowledge economy by contributing to the rise of the trans-disciplinary, practice-based knowledge generation.

In rural areas, it is important to observe how universities may combine global scientific discoveries to local co-production and exchange of knowledge (Charles 2016): as Goddard et al. (2016) recommend, the knowledge provided by the traditions and the values of rural communities should be considered when talking about sustainable development and innovation, as culture is part of the societal areas which the university could contribute to (Goddard et al. 2016). This is only one example of the many new roles of universities that were recently outlined by policy strategies for education and research, such as Europe 2020 (European Commission 2010) and that are central to the debate on the new Horizon Europe Research programme. In fact, as Mazzucato says (Mazzucato 2018), societal missions are complex because they are less clearly defined and indeed must be co-defined by many stakeholders. In particular, when these stakeholders belong to a specific community, the collaboration created to carry out context-based research, in order to answer to the needs revealed through the dialogue with them, is called Community-Academic Partnerships (CAPs) (Drahota et al. 2016). Similarly, there is collaboration with enterprises: University-Business Collaboration (European Commission 2011) refers to “all types of direct and indirect, personal and non-personal interactions between HEIs and business for reciprocal and mutual benefit, including collaboration in R&D, personnel mobility (academics, students and business professionals), commercialisation of R&D results, curriculum development and delivery, lifelong learning, entrepreneurship and governance”. HEIs can be crucial to mediate among sectoral, regional and national ecosystems of innovation, dynamically linking them to a variety of public and private actors and international institutions. This particularly applies to innovation in food systems, from local to global level: in this context, HEIs can bring together businesses, public bodies and representatives of the civic society to apply new methods and research paradigms in order to achieve some food-related Sustainable Development Goals (UN SDGs), such as “zero-hunger”. Climate change and, more recently, COVID-19 pandemic led to greater awareness of the need to create new and more sustainable patterns of consumption. Technological, social and policy innovations may help plan sustainable food systems that can create less pressure on natural resources and develop resilient food supply systems, which need to be constantly monitored, evaluated and, thus, improved (Brunori et al., 2020).

In a time of public scrutiny on how universities spend their funds, the aim of this work is to answer the following research question: “What are the main roles a University can play in rural areas?” The paper therefore provides a review of the main functions that HEIs may perform more in general in rural areas, by investigating the definitions and characteristics of some approaches such as Quadruple Helix of Innovation, the Civic University, University-Business Collaboration – UBC – and Community-Academic Partnerships – CAPs, as described in literature review. A longitudinal case study is then presented in order to give a concrete answer to the research question. It studies the specific case of a research team at the University of Macerata (Italy), a HEI located in a rural area of Marche Region, and their projects of civic involvement in the agri-food and tourism sectors, in order to investigate the role played by the institution at the local level. Moreover, the case study offers the opportunity to achieve a second objective, that is to outline some research questions and trajectories that could be addressed in the coming years by the international community of scientists investigating the complex dynamics of rural areas.

This paper is structured as follows: in the first part, a review of the literature on the civic role of HEIs in rural areas is presented, by highlighting the main strategies and tools adopted to achieve their third and fourth missions. In the second part, the longitudinal case study of the University of Macerata is described according to the frameworks of the Quadruple Helix of innovation, building on a taxonomy based on the UBC and to the CAPs. Findings and discussion are therefore outlined, and the conclusions highlight some key elements for further research.

## Literature review

## Quadruple Helix and Mode 3 Knowlege production frameworks

The Quadruple Helix and the Mode 3 systems (Carayannis and Campbell 2006, 2009) consider the strong role of universities in producing knowledge and creating innovation. The authors define innovation as the act of converting knowledge creation and production to knowledge application, diffusion and use. The concept of knowledge is therefore broadened as conceptualised by society, and it is thus identified also as a social process. The Quadruple Helix adds the fourth helix, the “public”, to the Triple Helix (Etzkowitz and Leydesdorff 2000) that already included the relationship between government, universities (higher education) and industry. According to this, the social processes of knowledge production include the culture and the values of a specific society (Carayannis and Campbell 2010). On this ground, Carayannis and Campbell (2006) proposed an advanced knowledge system (Mode 3) based on the integration of different knowledge and innovation modes through co-evolution, co-specialisation and co-opetition. Mode 3 presents the following characteristics, among the others:

- Pluralism and diversity, co-existence and co-evolution, and mutual cross-learning of different knowledge and innovation modes;
- Encouragement of interdisciplinary thinking and transdisciplinary application: hybrid thinking in different systems (e.g. “social ecosystem”); hybrid thinking and acting in different systems (e.g. “social ecology; “sustainable development”);
- Hybrid combination and/or use of different technologies.

Mode 3 promotes “creative learning” and a “creative co-evolution” by also cross-linking human rights, human development and the environment and thus opening to co-evolutionary learning (Carayannis & Campbell, 2010). When considering Quadruple Helix and Mode 3 within the context of regional development, it is worth to stress the importance of the vision about the future: each actor involved has its own vision concerning its own future and the whole region and, consequently, also its own strategies. In rural areas, the actors involved in Quadruple Helix take part to the process of regional development: they collaborate in a regional development network focusing on knowledge-intensive development. More specifically, the actors belong to regional public, semi-public, private and third sector organisations whose aim is to contribute to the development of the territory. These networks are usually informal, and the coordination is based on openness and reciprocity. In most cases, instead of a unilateral vision, it is possible to achieve shared visions about the region, by also emphasising the role of individuals (Kolehmainen et al., 2016). In order achieve these objectives, the actors involved need to undertake concrete actions, through both the creation and use of regional resources and competences, such as collective and collaborative activities based on regional knowledge (Kolehmainen et al., 2016). Lastly, universities spread moral values and reflective learning among students by involving them in a social level of engagement, related to the practical wisdom (Pitman et al., 2011). It is possible to talk about social innovation in terms of socially innovative practices: the civic university can play a transformative role in terms of change in the organisation of a social function to be collectively coordinated by new institutions able to change social power relations (Benneworth & Cunha, 2015).

This approach to innovation can also be applied to regional food systems in order to achieve sustainability. System and nexus approaches could be adopted to do it, also by integrating inter- and trans-disciplinarity. From this perspective, all the actors involved would operate in a system of relationships connecting activities, actors and outcomes, being aware that an impact in one sector may have consequences in others (Brunori et al., 2020).

### The civic university

According to Goddard & Kempton (2016: 1), in the civic university paradigm “teaching has a strong community involvement with the long-term objective of widening participation in higher education and producing well-rounded citizens as graduates”. Therefore, following the results of the dialogue with local stakeholders, HEIs should train future graduates who are able to take on the real challenges of the territory in terms of innovation. They may integrate teaching, research and engagement with the outside world, with each element supporting the other (Goddard & Kempton, 2016). Goddard & Kempton identify seven dimensions, as shown in Table 1.

**Table 1:** The civic university dimensions (Goddard et al., 2016)

Dimensions	Description
Sense of purpose	Creating an impact for society by addressing societal challenges or specific problems, both global and local. Creating benefits to defined groups, networks and communities and considering them as co-investigators and a source for knowledge.
Active engagement	Collaboration and dialogue to achieve social and economic development goals and enhance teaching and research. Internal collaborations: among academics in different disciplines. External collaborations: with other public and private organisations (education institutions, governments, business and cultural organisations).
Holistic approach	Engagement is an institution-wide activity that integrates the core activity of academics and enhances teaching and research. Students may benefit of it and be involved with the local community to improve knowledge, employability opportunities and active citizenship.
Sense of place	The civic university is well integrated within the territorial tissue where it is located: the place is a “living laboratory” providing specific opportunities to develop the work and impact.
Willingness to invest	Projects are built up to enhance the impact of research in universities beyond the academy and campus, by involving the academic and working staff in activities funded with internal or external resources.
Transparent and accountable	Civic responsibility: indicators and benchmarks to assess the performances, clear communication of its mission and vision and impact to stakeholders.
Innovative methodologies	Innovative methodologies and approaches to tackle societal challenges such as social innovation and entrepreneurship programs and collaborations among academics and academic and other organisations.

University can thus be conceived as an active agent able to create networks between local systems of knowledge and broader national and international circuits of knowledge and expertise (Atterton & Thompson, 2010).

As stressed by Riccaboni & Cavicchi (2019), policy strategies such as Europe 2020 (European Commission 2010) and European funding programmes, like the new Horizon Europe Research programme, highlight the contribution that HEIs may give to the mission towards societal changes. Many stakeholders take part in this process and HEIs may be mediators, playing the role of dynamic links between the sectoral, regional and national innovation ecosystems and the different public and private actors and international institutions (Mazzucato 2018). This can be true especially in the context of food systems, where a better policy coherence is needed in addition to better coordination and cooperation among several related sectors, including tourism and economic development (Réquier-Desjardins and Navarro 2016), and also to balance different needs and objectives.

HEIs are also recognised as pivotal players within The European Green Deal (European Commission, 2019b), and thus, in the context of the Next-Generation EU programme, for the COVID-19 pandemic recovery: collaboration “on climate change, sustainable energy, food for the future, and smart, environmentally friendly and integrated urban transport” among higher education institutions, research organizations and companies is promoted and supported by dedicated funding in order to achieve an ecological transition. Moreover, universities can play a role in developing and assessing knowledge, skills and attitudes on climate change and sustainable development by engaging with students and the wider community. Similarly, HEIs could contribute to the Farm to Fork strategy, for more sustainable food production and consumption. As they promote the use of sustainable practices towards a circular economy, technological innovation and the digital transformation, supporting the design of the strategic plans, they facilitate the dialogue among the stakeholders involved in the sector connecting them at a regional, national and international level.

#### University-business collaboration (UBC)

When it comes to university-business collaboration, this has to be created on solid grounds in order to solve different and complex problems: some challenges organisations have to face actually require capabilities they are not able to develop individually but only through the combination of multiple experiences, contexts and expertise (Beaver, 2004). In some cases, the same scholars need to move beyond their own research areas collaborating with other scholars from different disciplines, as to mutually benefit from an “outsider’s perspective” to recognise, by reciprocally sharing methods and approaches, their potential contribution in terms of novelty or to measure the reliability of their research (e.g. errors) (Beaver, 2004: 6). Sharing different logics, mindsets, skills and ideas leads to innovative thinking and allows new ideas to be generated. Moreover, the involvement of both parties in different collaborative research projects allows for the creation of a common ground on which further, wider research projects can be developed (Spekkink and Boons 2016). However, this also requires academics to develop competences that go beyond their own abilities in research: facilitation, consultancy, and project management skills are needed (Docherty and Smith 2007), as well as a more general relational attitude and interactional expertise (Bartunek 2007; Collins 2004). Consequently, from a collaborative perspective, it is important for academicians to be open-minded, ready to learn and also to change the course of the collaboration if new potentially relevant discoveries arise (Di Benedetto et al. 2019). In this way, academics are perceived as neutral by businesses: their interest is to provide publicly accountable results, acting with rigour, honestly (Docherty and Smith 2007).

#### Community-Academic Partnership (CAP)

When collaboration based on knowledge is mainly developed between universities and community members, the Community-Academic Partnership (CAP) model can be applied, which is “characterised by equitable control, a cause(s) that is primarily relevant to the community of interest, and specific aims to achieve a goal(s) (Drahota et al., 2016: 192). According to the model, community members (representatives or agencies) are involved who have knowledge of the cause, in addition to academic researchers.” Community-based participatory research and participatory action research have been usually applied in the context of CAPs with the purpose of reducing the academic-community stakeholders’ gap and providing benefits and interventions important to the community (Drahota et al., 2016). To better explain the collaborative processes underlying CAP development, a research model, the Model of Research-Community Partnership, has been used (Brookman-Frazee et al., 2012). In this model, relevance is given to the community context in which the collaboration process is developed and concretely sustains itself through actions and activities. Facilitating and hindering interpersonal and operational factors are therefore identified to start the process. Facilitating interpersonal factors are related to the quality of the relationships among partners:

- Trust and respect;
- The presence of shared visions and goals;
- Good communication (common language) and ability to solve conflicts;
- Clear division of roles and functions.

Hindering factors are opposite to the previous, also concerning different expectations about the results of the collaboration among partners.

Operational factors are related to the way the collaboration is actually managed. Facilitating factors refer to the quality of leadership and the choice of partners, the organisation of well-structured meetings and the resulting positive impacts on the community. Hindering factors refer to the partners’ perception on how consuming their commitment is in terms of time, funding pressures and control efforts, tasks and activities (Brookman-Frazee et al. 2012; Drahota et al. 2016).

The results of the process of collaboration may be different: they can be proximal (partnership synergy, knowledge exchange, tangible products) and distal (which depend on the proximal: development of/enhanced ability to implement programs or interventions, improved community care, sustainable CAP infrastructure for collaboration, changed community context) (Brookman-Frazee et al. 2012; Drahota et al. 2016).

## Methodology

The overarching aim of this study is to understand the roles that universities can play in a rural setting. A 10-year longitudinal interpretive case study (Yin 2003) is therefore presented to assess whether the frameworks of civic universities (Goddard et al. 2016), Quadruple Helix (Carayannis and Campbell 2006) and CAPs (Brookman-Frazee et al. 2012; Drahota et al. 2016) can be used to explain the multiple functions that the University of Macerata played in a time frame of 10 years (2009–2019). The action-research projects carried out by a group of researchers of the Department of Education, Tourism and Cultural Heritage of the University of Macerata are discussed. They focus on agri-food marketing, sustainable tourism, place branding and rural development, with a special focus on the learning needs of the territory in terms of management of culture and tourism.

## Background context

### The case of the University of Macerata

The University of Macerata (UNIMC) is located in in a rural area of Marche, in central Italy. It is one of the four public universities in the region has five Departments: Economics & Law; Law; *Political Science, Communication and International Relations*; and Education, Cultural Heritage and Tourism; Humanities – Languages, Language Liaison, History, Arts, Philosophy. It also offers three international master’s degree programs in English: International Tourism and Destination Management, International Finance and Economics, and Global Politics and International Relationships (https://www.unimc.it/en/courses/departments-and-schools. Accessed: September 20, 2020). In the academic year 2017/2018, 10,083 students enrolled at UNIMC and 438 of them were international students. In the same academic year, 467 people were employed at UNIMC, including full and associate professors and research fellows (MIUR 2020).

The region has always been described as a “plural” region, starting from its name, which is regarded as a plural noun opposed to all the other Italian regions. As Guido Piovene (1966) says, *Marches are a “distillate” of Italy*, being its landscapes and works of art the perfect, tiny representation of what Italy offers*.* The territory is characterised by “vertical stripes” from the inner to the outer, due to the presence of the Appennines, of the hills, of the valleys and then of the Adriatic coast. Thirteen rivers run parallel from the mountains to the sea, creating a so-called “comb” structure, which has affected the economic activities of the region, divided into industrial districts: these highly specialised areas mainly include family-run SMEs in the fashion, shoes, furniture and manufacturing industries.

According to the Regional Innovation Scoreboard (European Commission, 2019a), the financial and economic crisis that strongly hit the region since 2008 caused a deterioration in economic performances, investment propensity, employment opportunities and prospects. In particular, the crisis had a negative impact on firms and sectors that are less export-oriented. As it happened in many other Italian regions, at the same time, the fiscal consolidation measures taken at a national and regional level reduced the public resources available for regional development, thus resulting in a negative impact on disadvantaged territories and social groups (e.g. youths).

In terms of added value distribution, in 2016, the highest percentage was related to the service sector (68%), followed by the manufacturing and industry (30%) and agriculture (2%). In the same year, the agricultural sector employed 2.4% of the regional population, which is mainly employed in the service (61.4 %) and industry (30.7%) sectors (Marche Region 2018).

### Theoretical and methodological approaches

The University of Macerata identified the following strategies (UNIMC, 2018) to interact with the territory, in order to accomplish its third and fourth mission and to achieve an international dimension:

- Development of strategies for territorial marketing, by involving local actors to discuss and create contents;
- Strengthening of the fourth mission by reinforcing the idea of the university as a common good and as a public space where to build better the interactions with the city and the territory;
- Setting up of institutional round tables for discussion and planning, in order to reinforce the relationships among the city, the territory and the community;
- Building a network with key national and international actors to promote the territory;
- The creation of a responsive and dynamic institutional website containing all the information that can facilitate an integrated digital communication strategy.

In line with these goals, since 2009, a team of researchers from the Department of Education, Cultural Heritage and Tourism of the University of Macerata has been collaborating with local stakeholders on topics like territorial marketing, marketing of agri-food products, place branding and rural development. Their approach can be included in the field of action research (Gilmore and Carson 1996) and experiential learning (Kolb 1984). The first concept, action research, refers to the relationships among academics, professionals and stakeholders (Grant et al. 2001): based on their experience researchers investigate behaviours related to a phenomenon that involves specific stakeholders and then provide them with useful insights to develop entrepreneurial and managerial competencies. In the theoretical framework of experiential learning, the experience is relevant to gather information, learn something new or reinforce existing conclusions. It emphasises the role of experience in the learning process because it integrates experience, perception, cognition and behaviour (Kolb 1984, p. 21), thus providing holistic visions of a phenomenon.

Further aspects related to the methods applied by the research team have been analysed: the concept of *mutuality*, in which the relationship between the researcher and the stakeholders aims at creating reciprocal flows of communication and the concept of *commitment* with the personal involvement and coherence of the research design with the phenomenon observed (Cavicchi et al., 2014). Good relationships and trust among partners, which lead to shared visions and goals in order to provide operational steps and to pursue practical outcomes, characterise both CAPs and the Academician-Practitioner relationships (Brookman-Frazee et al. 2012; Cavicchi et al. 2014; Drahota et al. 2016).

Among the most applied methods that allow running researches to explore the real phenomenon and to provide educational services to students, the following can be mentioned: Problem-Based Learning and Open Space Technology (Owen, 2008).

In the first case, students are involved in the process as described by Barrows (2002, pp. 119–120):

1. “The problems are presented to the learner in the way they would present in the real world, as unresolved ill-structured problems, stimulating the generation of multiple hypotheses about cause and management”;
2. “The learners have to assume responsibility for their own learning, determine what it is they need to learn and the appropriate resources for the information from the world about them (texts, libraries, online, experts)”;
3. “The teacher’s role is that of a guide or facilitator of learning; commonly referred to in PBL as a tutor”;
4. “The problems chosen are those most apt to be confronted by the learner in life and career. The skills activities required of the learners are those valued in the real world—making PBL an authentic learning process”.

The Open Space Technology (Owen, 2008) is a bottom-up participatory approach in which participants are all involved in the discussion of a topic and responsible for their active participation and for the success of the meeting.

### The projects

**Fig. 1:**
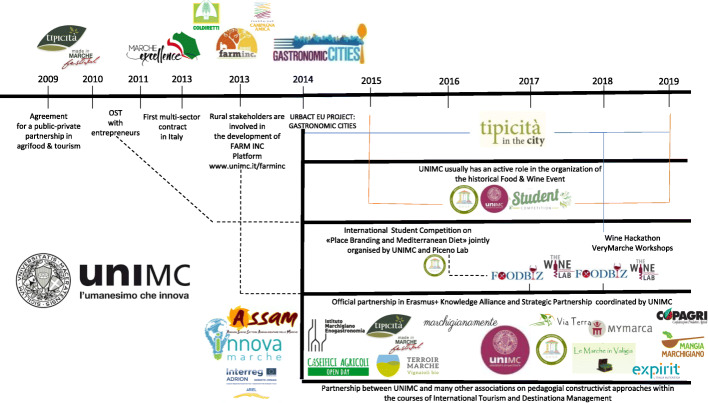
University of Macerata co-creation pathway (own elaboration)

A timeline describes graphically the several projects the team was involved in between 2009 and 2019 (Fig. 1), also showing the many other actors involved. In the following paragraph, a chronological description of the main projects is provided (Table 2).

**Table 2:** Overview about the projects representing UNIMC’s civic engagement (own elaboration)

**NAME OF THE PROJECT**	***Marche Excellence***	***Farm Inc***	***Gastronomic Cities***	***International Student Competition (ISC)***
**DURATION**	2009-2012 (ongoing network)	2013-15	2013-15	2015 - ongoing
**TYPE OF FUNDING**	Private-Public (private network)	Leonardo Lifelong Learning Programme	URBACT	Local public and private sponsorships/UNIMC internal funds
**DESCRIPTION**	**Initial Partnership**: Marche Region, University of Macerata, Banca Popolare di Ancona (UBI), Fermo Municipality (Tipicità Festival), Marche’s Chambers of Commerce. **Main goal:** creating a network among Made in Marche producers to promote the region. 2009: (During Tipicità Festival): Establishment of the Marche d’Eccellenza Forum, laboratory of ideas to promote the Made in Marche (food & wine and handicrafts); 2010: Marche d’Eccellenza Forum - discussion about the creation of a regional umbrella brand and tourism development (need for leadership/code of conduct) 2011: Marche d’Eccellenza Forum – no discussion, absence of Regional representatives, no regional umbrella brand. **Main outputs**: 2012: Marche d’Eccellenza contract network (13 entreprises) (Rinaldi and Cavicchi 2016)	**Partnership:** Italy, Greece, Belgium, Latvia, Cyprus. **UNIMC Financial Support:** € 50.149,00 (total project’s funding: € 297.971,00) **Main Goal:** facilitate the acquisition of skills by providing marketing training to the enterprises in the agri-food sector. **Main outputs:** online platform on marketing of agri-food products.	**Partnership:** Fermo (Marca Fermana Association) (FM), Alba Julia (Romania), L’hospitalet de Llobregat (Spain) and Korydallos (Greece). **Main goal:** promotion of gastronomy as a key for urban development taking Burgos -Spain (2015 UNESCO Citiy of Gastronomy) as a best practice. **Specific goal:** increase the reputation of Fermo as a cultural and gastronomic tourism destination. **Main outputs:** creation and implementation of a Local Action Plan for Fermo as a gastronomic tourism destination.	**Partnership:** UNIMC, Piceno Laboratory on Mediterranean Diet **UNIMC Financial Support:** Scholarship for the participation of 20 UNIMC students enrolled in International Courses (10.000,00 € in total per year) **Main goal:** giving value to the Mediterranean Diet as a leverage for the touristic development of Fermo Area. It consists in a contest among international students engaged in a dialogue with local stakeholder, in lectures on place branding and in experiential learning activities in order to provide innovative ideas for the promotion of the area and make an impact with a social media challenge (use of ICT) (Cavicchi et al. 2018). **Main outputs:** Increased visibility and reputation of the area on the social media; works and ideas provided by students.
**UNIMC TEAM INVOLVEMENT**	Provision of *super partes* expertise, facilitation of the discussion among participants and stakeholder engagement about the process for creating and managing the network. (Cavicchi et al. 2014).	Stakeholder engagement for the collection of learning needs; design, creation and provision of the learning materials; management of the web portal. Use as an online didactic tool for students.	Stakeholder engagement; participation in the elaboration of the Local Action Plan; research activities; Experiential learning activities involving students.	Co-creation of the initiative. Engagement of participants from international universities and from other regions (students and scholars); Involvement of UNIMC’s professors; Design of the didactic program (experiential learning (Kolb 1984); lectures; project-based/problem-based learning (Boud and Feletti 1997; Bell 2010; Blumenfeld et al. 1991)). Provision of scholarships for UNIMC students’participation.
**NAME OF THE PROJECT**	***ASSAM: Innovamarche and ARIEL***	***The Wine Lab - Generating Innovation between Practice and Research (TWL)***	***FOODBIZ - University and business learning for new employability paths in food and gastronomy***	
**DURATION**	*Innovamarche* 2016 – ongoing *Ariel* 2018-19	2016-2019	2016-2019	
**TYPE OF FUNDING**	*Innovamarche* EIP-AGRI (several sources: e.g. Horizon2020; ERDP) *Ariel* Interreg-Adrion (ERDF)	Erasmus+	Erasmus+	
**DESCRIPTION**	**Collaboration:** ASSAM (Regional Agency for Agri-food Services in Marche Region) – UNIMC **Stakeholders engaged**: researchers, policy makers, associations trade associations; local businesses. **INNOVAMARCHE** **Main goal:** creating a concrete support for the implementation of bottom-up innovative and sustainable projects and for connecting actors that could work together, get public funds and develop joint projects at national and European level in the agri-food sector. **Main outputs:** the creation of an online platform in which sharing all the innovative ideas and implementing them in operative groups, according to the specific needs identified on the territory and related to agriculture. **ARIEL** **Partnership:** Italy, Croatia, Greece and Montenegro. **Main goal:** promoting technological and non-technological solutions for innovation and sustainability in small-scale fishery and aquaculture in Adriatic-Ionian area through the creation of a knowledge network; through transferable activities. **Main outputs:** online platform to support transnational networking and permanent knowledge sharing.	**Partnership:** UNIMC (coordinator); universities, associations, policy makers, research centres and small wineries from Austria, Greece, Hungary, Italy and Cyprus (disadvantaged areas). **UNIMC Financial Support:** € 138.033,00 (total project’s funding: € 946.548,00) **Main goal:** stimulating knowledge flow, sharing challenges and solutions, and jointly generating and accelerating innovation in the wine sector; creation of hubs (clusters) as groups of interest and learning communities involving all the stakeholders interested in the sector. **Main outputs**: online platform for sharing knowledge, debate and provide learning materials to answer to common needs identified thanks to stakeholder engagement activities, towards the creation of wine hubs.	**Partnership:** UNIMC (coordinator). Other universities and associations from Italy, Sweden, Belgium, Spain, Croatia and Poland in the tourism, hospitality and agri-food fields. **UNIMC Financial Support:** € 65.761,00 (total project’s funding: € 276.136,00) **Main goal:** promoting the acquisition of employability skills in HEI’s students through their active involvement in context-based learning with local agri-food businesses. Promoting university-community co-creation for innovation and knowledge exchange. **Main outputs:** the FOODBIZ Handbook; the creation of learning communities; the production of free online learning resources and the creation of guidelines for other communities.	
**UNIMC TEAM INVOLVEMENT**	*INNOVAMARCHE* Support innovation brokering processes (Howells 2006) during the Info Days, acting as an intermediary for innovation among several actors. Facilitating the discussion (OST - Owen 2008) supporting the bottom-up creation of operative groups, helping in formulating innovative ideas, in looking for strategic partnerships, in designing project proposals. *ARIEL* Innovation brokering (OST – Owen 2008) for the facilitation of the discussion among local stakeholders for collecting learning needs (Ancona, 02/19). Design of a package of e-learning materials on marketing of fishery products, available for all the project partners. Participation to learning events for presenting the materials provided (Split, 06/19; Sicily, 10/19; Ancona, 12/19).	In the UBC perspective, in order to boost the process of cross-fertilisation: stakeholder engagement; organisation of participatory approaches to facilitate the dialogue among stakeholders and collect learning needs; organisation of experiential learning events (also involving students): wine hackathons, wine weeks (students and staff mobility); conferences, online contests (e.g. design a wine label). Design and provision of learning materials; management of the online platform; run researches.	Organisation of participatory experiential learning workshops, entrepreneurial discovery process events and conferences directly involving stakeholders and students. Collection of learning needs. Design of learning materials. Management of the online platform. Participation in the elaboration of the Handbook and Guidelines.	
**NAME OF THE PROJECT**	***Eureka*** ***PHD Programme*** ***for innovation***		***PBL in the classroom***	
**DURATION**	2012 - ongoing		2015 - ongoing	
**TYPE OF FUNDING**	ESF (ORP)		Internal funds	
**DESCRIPTION**	**Partnership**: UNIMC – Marche Region – Businesses **UNMC Financial Support**: UNIMC partially covers the PhD Scholarships (max 13.000,00 €per student) **Main goal**: providing co-funded scholarships for PhD innovation projects (50% time spent in a company). **Main outputs:** Applied research/R&D/PhD Thesis. Developing entrepreneurial skills in students. Implementation of UBC.		**Partnership:** Informal collaboration among UNIMC, local stakeholders, students. **Description:** stakeholders present their “challenges” during the *Agri-food marketing* and *Place Branding and Rural Development* classes: students work on real cases by applying their theoretical knowledge and competences so to provide suitable potential solutions (experiential learning and PBL (Kolb 1984; Barrows 2002) and to co-create new knowledge; develop employability skills. **Main outputs:** application of feasible solutions in the companies’ context; creating joint events and further projects; opportunities for students (internships, job positions, PhD scholarships).	
**UNIMC TEAM INVOLVEMENT**	Application for obtaining several scholarships within the Department of Education, Cultural Heritage and Tourism, in collaboration with local companies. Tutoring of PhD Students. Implementation of UBC.		Stakeholder engagement. Providing a theoretical background to students to work on real cases; tutoring students; stimulating discussion for knowledge exchange and co-creation. Discussing further opportunities of collaboration with stakeholders. Implementation of UBC.	

## Findings and discussion

In the following table (Table 3), the activities carried out by the University of Macerata as a part of the projects previously described are hereby compared to the civic university’s dimensions, in order to verify whether UNIMC can be considered a civic university.

**Table 3:** Self-assessment of University of Macerata activity (own elaboration)

PROJECTS		Marche Excellence	Farm INC	Gastronomic Cities	International Student Competition	Innovamarche/Ariel	The Wine Lab	Foodbiz	Eureka	Pbl (Classes)
**CIVIC UNIVERSITY DIMENSIONS**	**Sense of purpose**	X	X	X	X	X	X	X	X	X
	**Active engagement** (*Internal collaboration*)		X	X	X		X	X		
	**Active engagement** (*External collaboration*)	X	X	X	X	X	X	X	X	X
	**Holistic approach**	X		X	X		X	X	X	X
	**Students’ engagement**			X	X	X	X	X	X	X
	**Sense of place**	X	X	X	X	X	X	X	X	X
	**Willingness to invest**		X		X		X	X	X	
	**Trasparent & Accountable** (*Performance assessment*)		X	X	X	X	X	X	X	X
	**Transparent & Accountable** (*Vision/Mission Communication*)		X	X	X		X	X		
	**Transparent & Accountable** (*Impact assessment*)						X	X	X	
	**Innovative Methodologies**	X	X	X	X	X	X	X	X	X

Moreover, in the discussion, specific references to the Quadruple Helix, Mode 3 and CAPs frameworks are made.

### UNIMC as a civic university

The abovementioned projects show that since 2009 the University of Macerata has been acting with a sense of purpose and place. Its attitude to work and build a relationship with public and private stakeholders belonging to the Quadruple Helix is coherent with what Goddard & Kempton (2016) described as a civic university: the university achieved it through the combination of teaching and action research in order to face context-based challenges with the will to train “well-rounded citizens as graduates”. The territorial context for the development of the projects can be considered “the living laboratory” where putting into practice (Goddard & Kempton, 2016) all the activities designed in the projects. The projects involving UNIMC mainly focus on Marches Region’s rural area and are related to tourism destination management, place branding, marketing of local food and wine, sustainable tourism. The aim is to achieve societal challenges at a local level, generally related to sustainable rural development and to the concept of resilience connected to the territory after the consequences of the 2016 earthquake that hit Central Italy.

#### UNIMC’s holistic approach in an active engagement perspective: the role of facilitator

The University of Macerata, as a whole educational infrastructure, actively engaged in the projects: collaborations take place both internally and externally.

The projects were not always coordinated by the university, which, especially at the beginning, got involved thanks to the willingness of other actors (e.g. Marche Excellence, Gastronomic Cities/Urbact Programme). The university later gradually became a point of reference to facilitate discussions among participants. Most of the times, those discussions were useful to pursue the main objective to find a way to collaborate, to start “visioning among visions” (Kolehmainen et al. 2016) and to collect the actual needs of the actors involved, which helped to design strategic actions (Kolehmainen et al. 2016).

As in the Mode 3 model (Carayannis and Campbell 2006, 2009), all the projects were characterised by pluralism and diversity, being the actors’ needs, approaches and know-how usually different and the importance of individuality usually taken into account in the overall context of the project, besides collective result.

Through the application of stakeholder engagement methods, especially the OST one (Owen, 2008), the University of Macerata played a role in determining the possibility of co-existence and co-evolution among actors in connection with the objectives of the projects by stimulating, through discussion, mutual cross-learning of different knowledge and innovation modes (Carayannis and Campbell 2006, 2009). This could be directly observed in the *Marche Excellence*, *FarmInc*, *Gastronomic Cities*, *Innovamarche* and *Ariel*, *The Wine Lab (TWL)* and *FOODBIZ* projects. The knowledge could be transferred from the university to the other actors involved while the other actors could provide knowledge and data to the university and share them with other players’ belonging to other sectors, thus giving to the university the opportunity to research real phenomena and put into practice action research (Carayannis and Campbell 2006, 2009; Charles 2016; Goddard et al. 2016). This was possible also thanks to the quality of the relationships between researchers and stakeholders: trustworthiness, mutuality and commitment are the ground on which the partnerships involving the UNIMC’s research team has been developed since 2009. Most of the projects are characterised by the presence of a reciprocal flow of communication which led to shared visions, facilitating establishing shared goals and giving clear roles and tasks to each partner (Cavicchi et al. 2014; Brookman-Frazee et al. 2012; Drahota et al. 2016).

Those encounters helped to create additional formal and informal collaborations with both already existing partners and new ones (Spekkink & Boons, 2016), e.g. EUREKA (a kind of industrial PhD grants co-funded by companies and the regional government) or post-doc contracts; the presence of new local stakeholders at the PBL approach in the classroom and at events, with the active involvement of students contributing to the challenges presented by designing possible solutions; students volunteering in the initiatives proposed by local stakeholders (such as Caseifici Aperti or the Lavandaso Festival by Agrituraso), thus supporting the organisation and improving their learning activity through real experiences and also giving some feedbacks in terms of competences and knowledge.

Hindering factors (Drahota et al., 2016) for collaboration emerged in some cases, thus shaping the relationships among partners in a different way from the one previously expected: this could be the case of *Marche Excellence*, where the lack of public leadership led to the creation of a private network contract including 13 firms for the promotion of Marche region as a destination. In this case, the university also played a facilitating role, helping partners to discuss and understand which path they could follow.

##### Internal collaborations and the application of innovative methodologies

With regard to internal collaborations, researchers from several disciplines contributed to the implementation of FarmInc, Gastronomic Cities, International Student Competition, The Wine Lab and FOODBIZ projects: they shared their research methods and teaching approaches and proposed learning materials and workshops addressed to the stakeholders participating in the projects, which are developed considering them as co-investigators and a source for knowledge (Goddard and Kempton 2016). This also confirms the presence of a sense of purpose and place: the context and the real challenges identified through the dialogue with stakeholders created the basis to design project actions. From their experience in the projects, researchers from several fields were also able to carry out research on project-related topics (Gilmore and Carson 1996; Grant et al. 2001). Students were allowed to take an active part in cross-learning activities, especially during the Erasmus Plus projects and the ISC, with the aim of increasing their own employability skills and field-related knowledge. They could attend the workshops and further contribute to create learning materials and make their proposals based on the theory learned in the classroom and on the experience gained during the experiential learning activities (Kolb 1984; Barrows 2002). As a way of example, students’ engagement was part of the curricular activity during the Cultural Heritage Management course: students conducted interviews with small local producers about the role of Heritage Marketing in their historical firms, which became learning materials in TWL learning platform. In the same project, a scholar in history of images trained his students as guides in local art galleries, organising thematic tours about the symbolism of food and wine in the paintings. Moreover, both in TWL and FOODBIZ projects, some scholars from the Department of Education, Cultural Heritage and Tourism took part in the activities: Sociology researchers investigated the role of women as entrepreneurs in the wine sector and the relevance of intergenerational exchange and familiar bonds in the same field. The research was carried out in collaboration with a local association of sustainable winemakers, Terroir Marche, and the Wine Women Movement. A similar contribution was given by a professor in Informative Systems for Cultural Heritage, who organised with students several workshops on Wikipedia as a free tool for territorial storytelling based on relevant, verified historical sources: the practical workshops took place during events organised by local associations and within the funded projects, also as a part of the didactic program of the ISC in 2019. The workshop mainly focused on typical food and wine products. Students learned how to correctly write an encyclopaedia entry on Wikipedia, also providing a new source of information about the territory.

As a final act of both projects, in November 2019 researchers from different departments organised an international conference about food and wine in Macerata, inviting colleagues and scholars from other Italian and international universities: the conference was another opportunity to combine several disciplines, such as Literature, History of Art, Cultural Heritage Management, Geography, Economics, Territorial Marketing, Entrepreneurship, in a discussion about representations, cultural identities and the co-creation of sustainable development. Students and PhD students presented the results of their studies in those fields.

Even though these examples showed a successful interdisciplinary approach, proving the ability to collaborate among the different disciplines, each one of them remaining linked to its own field of knowledge. Such an approach is useful to gain awareness about novelty or potential errors (Beaver, 2004). However, an effort could be done to take a step towards innovation in knowledge co-creation, by shifting to a transdisciplinary perspective, where an integration among subjects could be created by overcoming boundaries and by considering knowledge as a whole, in a non-fragmentary way.

##### External collaborations and the application of innovative methodologies

Over the years, UNIMC has created networks at a local level and also gradually broadened national and international circuits of knowledge and expertise (Atterton and Thompson 2010). All these are based on a relational attitude (Docherty and Smith 2007) which expresses itself through openness to learn from each other and interact (Di Benedetto et al. 2019). Investigating and discussing similar situations in other countries could contribute to tackle local societal challenges and find innovative solutions that may be adjusted to local contexts. Vice-versa, the solutions proposed locally could work as a source of inspirations for other contexts. From this idea of exchange and sharing, external informal collaborations were created with other universities at a regional, national and international level with scholars who have a similar view and commitment to face real world challenges.

At a local level, for example, ICT engineers from Marche Polytechnic University (UNIVPM) developed apps to be tested and software to monitor social challenges during all the editions of the ISC, since 2015. More recently, UNIMC and UNIVPM, started working together on the idea developed by a local entrepreneur, Mangia Locale, which is an App that promotes a network of local farms selling their products to direct consumers. They also jointly designed a solution for home delivery during COVID-19 emergency. Through this project, an R&D post-doc contract was funded. It confirms what Spekkink & Boons (2016) say about collaboration: it helps to create a common ground fostering co-working in further research projects.

International collaborations exist and are mainly based on trust and good relationships among partners (Brookman-Frazee et al. 2012; Drahota et al. 2016): this is true, for example, for the international universities taking part to the ISC and for the international consortia of EU funded projects.

External local collaborations are the base of all the projects. Private and public stakeholders are involved in order to improve the coherence between the projects’ general goals and the potential impact on the territory (Goddard & Kempton, 2016): in most cases, regional and local authorities, trade associations, cultural associations for the promotion of the territory, cultural institutions and firms located in Marches’ rural areas play an active role in the activities designed. Those encounters, sometimes, help to create stronger relationships based on trust and exchange and thus lead to further ideas for joint projects and create opportunities for students to keep on working on real cases (also through official agreements for curricular and extra-curricular traineeships) (Spekkink & Boons, 2016).

Innovative methodologies and approaches are applied to tackle societal challenges: participatory approaches and experiential learning activities are usually adopted (Goddard & Kempton, 2016) with the aim to pursue social innovation and promote an entrepreneurial mindset among the participants. The research team usually act as a *super partes* facilitator, particularly in the different innovation brokering projects, in which the aim of researchers was to stimulate the discussion among local stakeholders in the agri-food and fishery sectors (Innovamarche and Ariel) and Gastronomic Cities, as to support the creation of a Local Action Plans.

Through external collaborations, often related to European funded projects, the University of Macerata acts as a dynamic link between sectoral, regional and national innovation ecosystems and different public and private actors and international institutions (Mazzucato 2018), especially in the food sector (Réquier-Desjardins and Navarro 2016), starting from the rural areas of Marches Region. These projects potentially support this role, by linking the local context to the global one and by creating internal links, also locally, among different sectors (Réquier-Desjardins and Navarro 2016). European programmes and policy strategies on sustainable development (e.g. Europe2020; Horizon2020; the European Green Deal, etc.) provide guidelines that allow the local level to be aligned to the global one and, vice-versa, local experiences to potentially become a best-practice at an international level, in other local contexts, as to work towards a more sustainable society.

#### UNIMC promoting knowledge transfer

Co-learning and knowledge circulation sometimes led to interdisciplinary thinking, in a process towards the creation of a transdisciplinary mindset, useful to work out practical solutions to real problems (Carayannis and Campbell 2006, 2009; Rinaldi et al. 2018). In the case here presented, this process includes the discussion among local actors, the active engagement of students, the identification of learning needs and, consequently, the implementation of action research (Gilmore and Carson 1996; Grant et al. 2001).

This may be the case of Innovamarche and Ariel projects, coordinated by the Regional Agency for Agri-food Sector Services of the Marche Region (ASSAM), in which the university was involved to find innovative solutions for agri-food businesses. In the case of Innovamarche, the university connected businesses with researchers who could help them to apply ICT to their needs. In the Ariel project, small scale fisheries and aquaculture businesses in the Adriatic Sea were provided with marketing elements for the promotion and communication of sustainability in their sector, by developing hybrid thinking and promoting knowledge exchange (Carayannis and Campbell 2006, 2009) thanks to the contribution of the university in terms of stakeholder engagement and creation learning material based on the results of the discussion.

Several events were directly organised by the university itself by applying the OST methodology (Owen, 2008). Local stakeholders from several fields, such as tourism and hospitality, agriculture and wine and gastronomy, culture and education, shared their visions and approaches on the future of Marches Region, submitting concrete proposals and ideas and, in some cases, considering to jointly pursue similar objectives for local sustainable development. Students usually took an active part in the discussion. One of these events was organised in January 2017 to discuss what impact the earthquake that hit central Italy in 2016 had on the Marches’ environment, economy and society, as to propose some hypothesis for reconstruction. Other participatory events (e.g. experiential learning workshops; entrepreneurial discovery process events) took place within funded projects, such as TWL and FOODBIZ. Researchers, practitioners and students from specialised sectors, such as Food & Wine and Tourism, guided the co-creation pathway in an international context (Trencher et al., 2014), by sharing their diverse competencies and methodological approaches in the fields. The projects’ objectives were innovation, knowledge creation/exchange and employability. Both projects prepared the ground for the implementation of a further objective: to turn the University into a hub, an interactive learning community, a structured point of reference in the territory where participants from the Quadruple Helix can meet, discuss, share knowledge, *contaminate*, co-design actions for the sustainable development of rural areas, and broaden their network. This could be seen as a distal outcome emerging from collaboration, as the creation of a sustainable infrastructure for CAP (Brookman-Frazee et al. 2012; Drahota et al. 2016).

The ISC combined the use of different technologies with a transdisciplinary approach (Carayannis and Campbell 2006, 2009) related to experiential learning (Kolb 1984) and PBL (Barrows 2002).. The competition was, thus, conceived as:

- An innovative way for students to learn within a context and through the dialogue with local actors belonging to the Quadruple Helix model. Students had to face real-world challenges being supported by their teachers as tutors; they autonomously led their learning to acquire skills useful to their professional career;
- A tourism attractor;
- A way to provide the territory with the immediate perceptions of foreigner visitors about local hospitality, also through the use of ICT and social media for place branding.

#### UNIMC’s willingness to invest: transparency and accountability

As UNIMC is involved in action context-based research, the goal is to have an impact beyond the academy (Goddard & Kempton, 2016). UNIMC thus make investments through the involvement of academic and working staff in activities funded with internal or external resources. Investments depend, first of all, on public funding. The projects, whether they are coordinated or participated by a research team, are usually funded by European programmes. The group, having also developed skills in project management, is active in applying to official calls for proposals to pursue action-research objectives (Docherty & Smith, 2007). It happened with TWL and FOODBIZ cases, funded by the Erasmus Plus Programme. Through these funds and with the support of public-private partnerships, as for the case of the ISC, the team promote stakeholder engagement activities aiming at facing specific challenges at a local level, whose results can be shared with other international partners. Research activities are also carried out thanks to private financial contributions: this is the case of the EUREKA programme, where local businesses provide partial funding for 3-year PhD scholarships in order to benefit from the presence of the candidates in the company and from the results of a shared research project.

Nevertheless, there are some limitations in the measurements of the impacts. If a major impact emerged, it is related to the creation of a network that was able to expand and grow in self-awareness. Specific indicators are needed to measure this aspect, such as the number of stakeholders involved in the network and the number of projects co-created with UNIMC since 2009, but also the number of projects developed thanks to the facilitating role of the university, without the university itself as a partner. It is more difficult to assess an economic or social impact on the territory due to the lack of perception about the active engagement of the university at a local level outside of the network. As part of its civic responsibility (Goddard & Kempton, 2016), UNIMC should provide an evaluation strategy and indicators to assess its performances and clearly communicate the mission and vision related to the projects and the results obtained to the stakeholders and, more in general, to the civic society not directly involved in the projects. Some of these indicators could be formulated on the basis of the outcomes that CAPs are supposed to create (Brookman-Frazee et al. 2012; Drahota et al. 2016). However, while such a task is easier when applied to European funded projects, which identified quantitative and qualitative indicators for a given set of outputs and outcomes, it is less applicable when it comes to informal collaborations. First, this is due to a lack of metrics to assess the impact of community engagement (MacQueen et al. 2015; Esmail et al. 2015), which is a crucial component of the CAP. Furthermore, while university works within a multi-layered strategy, which includes research, education, and its civic role, the specific outputs and outcomes are redefined continuously through the dialogue with the community; therefore, it is actually difficult to set clear performance indicators in the beginning as they require constant revision. In addition to that, the qualitative component of the CAP is essential, but its assessment is based on partners’ perceptions (McNall et al., 2009), which do not ensure the effectiveness of a measurable benefit for local economy and society.

## Conclusions

Nowadays, new challenges are arising for universities. HEIs are called to reconsider their role in society and their contribution to regional, economic, social and cultural development (Cavicchi et al. 2013). Globalisation and regionalisation processes are running together, and universities have the responsibility to combine different levels of knowledge by developing different attitudes and skills for their researchers. Universities can be the centre of local and regional learning and innovating partnerships, connecting different partners, creating a sustainable learning organisation and developing on-going leadership capacity in the region (Rinaldi et al. 2018). From this point of view, HEIs may also play a role in the recovery from the COVID-19 pandemic, meeting challenges that can lead to a more sustainable society. This role is highlighted in the European Green Deal (European Commission 2019b), and therefore, in the context of the Next-Generation EU, with dedicated funding to achieve the programme’s goals: collaborative research for ecological transition; civic engagement with students and within the local social context to develop and assess sustainability-related knowledge, skills and attitudes; facilitation of dialogue to support strategic planning for sustainable food systems transversally at regional, national and international level (e.g. Farm to Fork Strategy).

The case of the University of Macerata shows that even a small university located in a rural area characterised by a traditional economic sector can become a “multi-stakeholder platform engaged with society in a continual and mutual process of creation and transformation” (Trencher et al., 2014, p. 8).

Nevertheless, having the agri-food sector a strong relevance for the sustainable development of rural areas, also in connection with other sectors (e.g., tourism), HEIs, and in this specific case, the University of Macerata, can play a role in the systematisation of the relationships among actors, activities and outcomes and in the evaluation and monitoring of the chain of impacts within the different sectors that can be involved (Brunori et al., 2020). The challenge of sustainability at a regional level, in rural areas, can be more easily met by giving value to local excellences and to the existing social networks within the territory, which sometimes were created as a resilient reaction to difficulties (e.g., Central Italy Earthquake in 2016). In addition to the engagement of all the actors of the Quadruple Helix, in order to develop technological, social, and policy innovations useful in planning sustainable and resilient food supply systems (Brunori et al., 2020), it may also be important to encourage internal collaborations with academics from the same university or from universities belonging to the same region (external collaborations at a local level), who are committed in different research areas and could cooperate to create and implement joint projects, based on regional knowledge resources and competences (Kolehmainen et al., 2016). It could be helpful to shift from an interdisciplinary approach towards a transdisciplinary perspective as to integrate approaches, methods and themes to successfully co-create innovation. This aspect could also help to further develop the potential role of the University of Macerata in creating a sustainable place-based learning system for regional leadership capacity building (Brookman-Frazee et al. 2012; Rinaldi et al. 2018). In terms of transparency and accountability (Goddard et al. 2016), further research could be carried out to assess the projects’ performance and impacts, perhaps using sustainability as a lens of analysis in order to consider its social, economic and environmental dimensions. What becomes evident from this recognition, but should be actually measured, is the “snowballing” effect in terms of networking opportunities: the creation of a more organic infrastructure, that is a hub, within the University of Macerata, could be useful to create awareness in the community about the university openness to the territory and could allow the design of more structured activities and a more detailed plan to measure performance and impact with the identification of specific indicators.

## Data Availability

Not applicable

## Abbreviations

ASSAM: Regional Agency for Agri-food Services in Marche Region
CAPs: Community-Academic Partnerships
HEI: Higher Education Institution
ICT: Information and Communication Technology
ISC: International Student Competition
PBL: Problem-based learning
R&D: Research and Development
SDGs: Sustainable Development Goals
TWL: The Wine Lab
UBC: University-business collaboration
UNIMC: University of Macerata
UNIVPM: Marche Polytechnic University
